# Identification and full genome sequencing of previously unknown sandfly-borne phleboviruses using a newly established capture-based next-generation sequencing approach

**DOI:** 10.1128/jcm.01082-25

**Published:** 2026-03-13

**Authors:** Edwin O. Ogola, Inga Slothouwer, Gilbert Rotich, Anne Kopp, Armanda D. S. Bastos, Caroline Getugi, Julia Melchert, Terence C. Jones, Dorcus C. A. Omoga, Rosemary Sang, Baldwyn Torto, David P. Tchouassi, Sandra Junglen

**Affiliations:** 1 International Centre of Insect Physiology and Ecology (icipe), Nairobi, Kenya; 2 Department of Zoology and Entomology, University of Pretoria 56410, Pretoria, South Africa; 3 Institute of Virology, Charité Universitätsmedizin Berlin, Corporate Member of Free University Berlin and Humboldt-University Berlin, Berlin, Germany; 4 German Centre for Infection Research (DZIF), Partner Site Charité, Berlin, Germany; 5 Centre for Pathogen Evolution, Department of Zoology, University of Cambridge 2152, Cambridge, United Kingdom; Wadsworth Center - NYSDOH, Albany, New York, USA

**Keywords:** sandfly, phlebovirus, capture-based NGS, arbovirus surveillance

## Abstract

Sandfly-borne phleboviruses cause febrile illness and neuroinvasive disease in humans. While infections are reported in the Mediterranean region, the discovery of previously unknown phleboviruses in sandflies from Kenya suggests a wider geographic distribution. Detection and characterization of novel phleboviruses are often hindered by low-quality and low-viral-load samples. We developed a capture-based target enrichment next-generation sequencing approach that showed a 99%–100% fold enrichment of viral genomes from primary material and provides a robust tool for generating complete genomes of both known and previously unknown viruses. From a collection of 15,652 sandflies in Kenya, we recovered seven complete coding sequences of Embossos, Bogoria, and Kiborgoch viruses, and of two previously unknown phleboviruses, which were named Sosoik and Shable viruses. Sosoik virus shared 83% amino acid identity in its RdRp gene with that of Bogoria virus, while Shable virus shared ca. 88% amino acid identity with viruses of the Salehabad serocomplex. Additionally, a reassortant of Shable virus was detected that possessed an M segment from an undescribed Ponticelli-like virus. DNA barcoding of blood-fed sandflies revealed several potentially novel *Sergentomyia* species and evidence of host-feeding on humans, livestock, and reptiles, suggesting possibilities for zoonotic transmission. Overall, our findings increase the known genetic diversity of Old World sandfly-borne phlebovirus species from 18 to 25 (by 38.9%), including the detection of viruses from all pathogenic sandfly-borne phlebovirus serocomplexes in East Africa, opening new horizons in disease ecology research.

**IMPORTANCE:**

Knowledge of the genetic diversity of circulating pathogens is crucial for providing appropriate diagnostics and disease management. This study established a novel capture-based target enrichment next-generation sequencing approach that enabled the near-complete viral genome recovery from primary samples, while native NGS yielded negative or poor-quality results. In addition to the five recently discovered sandfly-borne phleboviruses in Kenya, two previously unknown phleboviruses were detected in sandflies from the same region. The viruses were detected in several sandfly species, which showed diverse host-feeding behaviors, including mixed feeding on humans and chickens. The study significantly advances the understanding of sandfly-borne phleboviruses by uncovering their broader geographic distribution and genetic diversity, particularly in East Africa, highlighting the importance of expanding surveillance efforts beyond traditionally studied regions.

## INTRODUCTION

Sandfly-borne phleboviruses can cause febrile illness and neurological disease in humans (1–5). The disease, commonly known as sandfly fever or phlebotomus fever, is endemic in the Mediterranean region. The recent discovery of previously unknown sandfly-borne phleboviruses in East Africa corroborates earlier serological evidence and suggests that the disease also affects African countries (1, 4, 6–8). Knowledge of the geographic distribution and diagnostics of sandfly-borne phleboviruses is limited. The viruses exhibit substantial genetic diversity, and their primary isolation in cell culture is challenging (7, 9). Traditionally, the characterization and genome sequencing of novel sandfly-borne phleboviruses have relied exclusively on material derived from infectious cell cultures (10–18). Consequently, the discovery of disease-causing agents was limited to viruses that could be isolated in cell culture. Improved sequencing techniques are essential to enhance diagnostic accuracy and advance our understanding of disease induced by sandfly-borne phleboviruses. There is a critical need for new methods to sequence and characterize these viruses, as well as to diagnose fevers of unknown origin appropriately in areas where sandflies are common. In particular, next-generation sequencing (NGS) approaches using targeted bait capture have not yet been applied to phleboviruses and represent a promising but unexplored strategy for advancing research in this field.

Sandfly-borne phleboviruses of medical importance belong to three serocomplexes. Sandfly fever Naples virus (*Phlebovirus napoliense,* SFNV) and Sandfly fever Sicilian virus (*Phlebovirus siciliaense,* SFSV) cause a self-limiting febrile illness and occur as sporadic outbreaks in the Mediterranean region (19–28). Toscana virus (*Phlebovirus toscanaense*, TOSV), a member of the Sandfly fever Naples serocomplex, is the leading cause of neuroinvasive infections, such as meningitis and encephalitis, in Italy (20, 29–31). Infections can lead to peripheral neuropathy, such as para- and hyperesthesia, myositis, fasciitis, deafness, facial paralysis, Guillain-Barré syndrome, and hydrocephalus, including coma (2). Sandfly fever Turkey virus is related to SFSV and has been identified as a causative agent for viral encephalitis in Turkey (32). Adria virus, a phlebovirus within the Salehabad serocomplex originally isolated in Albania, is associated with febrile illness in Greece (33). Sandfly-borne phleboviruses occur in the New World (North, Central, and South America) and in endemic regions of the Old World (Mediterranean area, North and East Africa, the Indian subcontinent, and Asia) (27, 28). Five serocomplexes, containing 23 sandfly-borne phlebovirus species, have been described in the Old World, namely the Sandfly fever Naples, Sandfly fever Sicilian, Salehabad, and Karimabad serocomplexes, as well as the recently described Marigat serocomplex (7, 17, 34).

Due to the limited surveillance of sandfly-borne phlebovirus infections and the challenges of virus detection in primary material, such as human serum (short viremia, low viral load) (35, 36), human disease caused by sandfly-borne phleboviruses is presumably underreported, especially in developing countries (4). Knowledge of the geographical distribution and genetic diversity of sandfly fever viruses has increased over the past few decades, particularly owing to advanced technological approaches, such as NGS (10–18). Nevertheless, detection and genome sequencing of novel viruses can be challenging due to sequence diversity and low amounts of viral nucleic acids in primary material. NGS is often used in combination with culture-based approaches to overcome low concentrations of nucleic acids (37, 38). Capture-based target enrichment NGS is used to selectively capture and amplify viral target sequences from samples with low viral load in order to recover complete genomes at high coverage (39). Target enrichment methods use commercially designed probes such as biotinylated DNA or RNA baits for in-solution methods or immobilized microarray baits for on-array methods (40). The baits are designed based on existing viral reference genomes, and sequences with nucleotide (nt) divergence of up to 40% to reference baits can be obtained from diagnostic samples (41). These methods allow for the detection of previously unknown viruses and have been successfully used to expand the knowledge on viral genetic diversity, for example, of coronaviruses and hepatitis E virus (41, 42).

Phleboviruses (family *Phenuiviridae*, order *Bunyavirales*) are enveloped, single-stranded negative-sense RNA viruses with a tripartite genome (43). The L segment encodes the RNA-dependent RNA polymerase (RdRp), the M segment encodes the glycoprotein precursor complex (GPC; consisting of Gn and Gc) and a non-structural protein (NSm), and the S segment, with two ambisense open reading frames, encodes the nucleocapsid protein (N) and a non-structural protein (NSs) (44). The International Committee on Taxonomy of Viruses (ICTV) has classified 82 phlebovirus species, of which 45 have been detected in sandflies (44, 45).

Human infections with sandfly fever viruses have been clinically reported since the end of the 19th century in the Mediterranean, North Africa, the Middle East, and central Asia (46–54). However, evidence for sandfly-borne phlebovirus infection in humans in sub-Saharan Africa is limited. Studies from the 1970s and 1980s found seroreactivity against Sicilian and Naples viruses in human serum samples from Somalia, Sudan, and Ethiopia, but viral sequence information was not obtained (55). Neutralizing antibodies against Ntepes virus (*Phlebovirus ntepesense,* NTPV) have been found in humans in different regions of Kenya, but it is unclear whether the virus causes symptoms of disease in humans (6). Several previously unknown sandfly-borne phleboviruses have been detected in sandflies from Kenya, namely Kiborgoch virus (*Phlebovirus kiborgochense,* KBGV), a member of the Sandfly fever Naples serocomplex, and Bogoria virus (*Phlebovirus bogoriaense,* BGRV), Embossos virus (*Phlebovirus embossosense,* EMBV), and Perkerra virus (*Phlebovirus pekarrense,* PERV), that form the Marigat serocomplex (7). BGRV, EMBV, and PERV define a highly diversified monophyletic clade in sister relationship with the Sandfly fever Sicilian serocomplex. Since KBGV, BGRV, EMBV, and PERV are related to the medically important SFNV and SFSV, they may induce similar symptoms of disease as their relatives (7). The geographical distribution, pathogenicity, and genetic diversity of these viruses remain to be fully understood.

The aim of this study was to develop and evaluate a capture-based NGS approach for whole-genome sequencing of sandfly-borne phleboviruses, in order to expand the knowledge of their genetic diversity. The newly developed bait set was successfully used to sequence the complete genomes of previously unknown phleboviruses detected in sandfly primary material from Kenya.

## MATERIALS AND METHODS

### Design of a myBaits set for phleboviruses

For specific in-solution hybrid capture and enrichment of phlebovirus sequences in NGS libraries, a myBaits set (Arbor Bioscience, Ann Arbor, MI, USA, obtained from BioCat, Heidelberg, Germany) was custom-designed based on the coding sequences from 19 sandfly-borne phlebovirus species (Table S1). In total, 57 complete genome sequences were retrieved from GenBank, aligned, and soft-masked for simple and low-complexity repeats by the company (Arbor Bioscience, Ann Arbor, MI, USA). Based on these sequences, 80 nt baits were designed and BLAST-filtered against the host genomes of *Phlebotomus papatasi, Phlebotomus chinensis, Lutzomyia longipalpis,* and *Nyssomyia umratilis,* resulting in a final design of 8,264 baits (sequences available upon request).

### Quantification of infectious particles (50% tissue culture infectious dose [TCID_50_] assay)

Virus stocks of 11 sandfly-borne phleboviruses obtained from the European Virus Archive Global were used as reference viruses for the evaluation of the bait set (Table S2). Vero E6 cells were inoculated with 25 µL of reconstituted lyophilizate of each virus, incubated at 37°C in 5% CO_2,_ and harvested after 2 to 10 days upon observation of virus-induced cytopathic effect (CPE). To produce viral stocks, Vero E6 cells were inoculated with 50 µL of each virus in a T25 flask. Cells were incubated at 37°C with 5% CO_2_, monitored daily for a maximum of 11 days, and harvested upon development of CPE.

The number of infectious particles of all strains was determined by TCID_50_ assay (56). Twelve 10-fold serial dilutions of the stocks were prepared in Dulbecco’s modified Eagle medium. Vero E6 cells were inoculated with 100 µL of each dilution in 96-well cell culture plates, incubated at 37°C with 5% CO_2_, monitored daily, and evaluated upon development of CPE. Stocks were stored at −80°C for further use.

### Native whole-genome sequencing

Number of infectious particles was normalized to 10^3^ TCID_50_ mL^−1^ for each reference virus stock and used for extraction of RNA. RNA was extracted using the NucleoSpin RNA Virus Kit (Macherey & Nagel, Düren, Germany) following the manufacturer’s protocol. Libraries of extracted RNA were prepared using the KAPA RNA HyperPrep Kit for Illumina sequencing (Roche Diagnostics, Mannheim, Germany) following the manufacturer’s protocol. RNA fragmentation was done at 85°C for 6 minutes. After cDNA synthesis, adapter ligation, and bead-based cleanup, library amplification was done in 12 amplification cycles due to initial RNA concentrations of <5 ng/µL, using the 2× KAPA HiFi HotStart Ready Mix and P5/P7 primer mix provided in the kit. Library concentrations were measured using the Qubit Fluorometer (Thermo Fisher Scientific, Waltham, MA, USA), and fragment sizes were identified using the 4200 TapeStation system (Agilent Technologies, Santa Clara, CA, USA). After normalization, the libraries were pooled and paired-end sequenced on MiSeq and NextSeq platforms, with a read length of 250 and 150 base pairs (bp), respectively (Illumina, San Diego, CA, USA).

### Capture-based target enrichment whole-genome sequencing

Sequencing libraries of extracted RNA were prepared for sequencing as described above. The final libraries were grouped into pools of four or five libraries according to their sequence divergence from the bait target. The library pools were enriched using the newly designed myBaits in-solution hybrid capture target enrichment system for phleboviruses as described above, following the manufacturer’s protocol (57).

Hybridization temperature was set to 60°C, and the same temperature was used for bead washing. Amplification of the captured libraries was done in 20 amplification cycles using the 2× KAPA HiFi HotStart Ready Mix and the P5/P7 primer mix provided with the KAPA RNA HyperPrep Kit for Illumina sequencing (Roche Diagnostics, Mannheim, Germany). After amplification, the enriched libraries were purified using Kapa PureBeads from the kit. Pool concentrations were measured using the Qubit Fluorometer (Thermo Fisher Scientific Inc.) and then normalized before paired-end sequencing on MiniSeq and MiSeq platforms, with a read length of 150 and 250 bp–300 bp, respectively (Illumina, San Diego, CA, USA).

### Sandfly collection and identification

**Fig 1:**
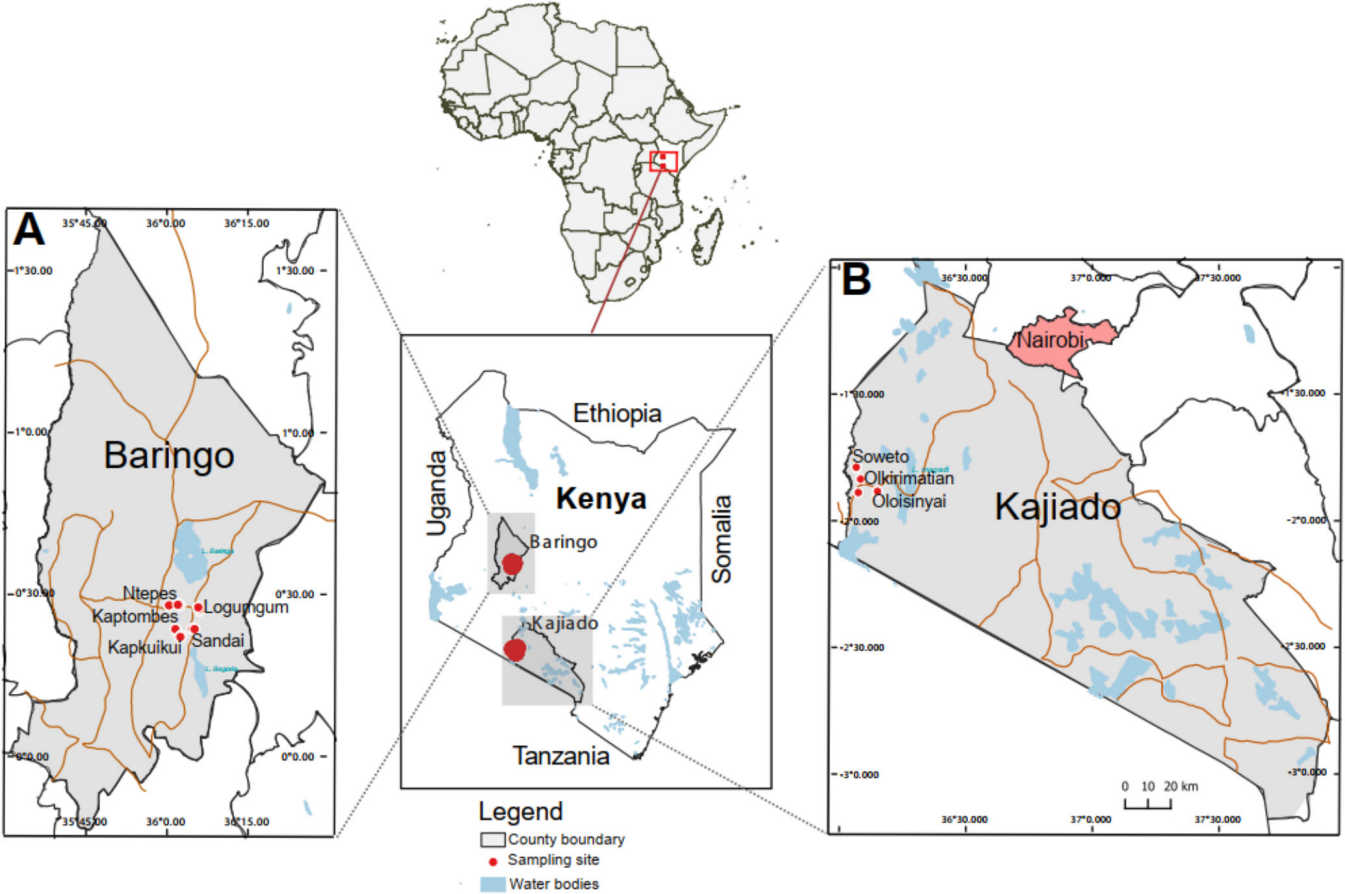
The geographical location of sandfly collection sites. Sandflies were collected in two counties, Baringo (**A**) and Kajiado (**B**), in Kenya. Sites of collection indicated by red dots. The map was generated in QGIS version 2.12 (open-source geographic information system) (59) using GPS coordinates and shapefiles from Natural Earth and Africa Open Data (Kenya counties shapefile; Creative Commons license). The shapefiles were downloaded on 20 October 2021.

Sandflies were collected between August 2019 and July 2020 in Baringo and Kajiado counties, two semi-arid to arid regions of Kenya. Details of sampling sites are described elsewhere (58). In Baringo County, sampling was conducted at five sites, namely Kapkuikui, Kaptombes, Logumgum, Ntepes, and Sandai, while in Kajiado County, sampling was conducted at three sites, namely Olkirimatian Conservancy, Oloisinyai, and Soweto (Fig. 1).

Adult sandflies were captured outdoors and indoors for 8 consecutive nights from 18:00 h to 06:00 h the following day in August 2019 and January 2020 in Baringo County, and in July 2020 in Kajiado County, using CDC miniature light traps (CDC-LT, model 512, John W. Hock Company, Gainesville, USA) (60). Trapped specimens were anesthetized with triethylamine, temporarily stored in liquid nitrogen in the field, and then at −80°C in the laboratory at the International Centre of Insect Physiology and Ecology (*icipe*), Nairobi. To increase the chances of virus detection and isolation, temperature changes were avoided by reducing handling time, and the collected sandflies were not identified at the species level or sexed (61). Based on trapping site, collection date, and abdominal status, samples of unfed sandflies were analyzed in pools of 1 to 50, while blood-fed specimens were analyzed individually.

### Blood-meal source and species identification of engorged sandflies

Sandflies were homogenized in Dulbecco’s phosphate-buffered saline (pH 7.4), and the homogenate was centrifuged in a benchtop centrifuge (Eppendorf, USA) at 2,500 revolutions per minute for 10 min. The clarified supernatant was used for virus isolation and PCR-based screening. For individual blood-fed specimens, pellets were preserved for blood-meal source determination and sandfly species identification.

The DNeasy Blood and Tissue Kit (Qiagen, Hilden, Germany) was used to extract DNA from pellets according to the manufacturer’s instructions. Vertebrate blood-meal source identification involved PCR-based amplification of the cytochrome c oxidase subunit 1 (*COI*) fragment as previously described (62). Molecular identification of sandfly species involved amplification of the *cox1* barcoding region using an established protocol, as previously described (63). PCR products were purified using ExoSAP-IT (USB Corporation, Cleveland, OH, USA) and submitted for Sanger sequencing (Microsynth Seqlab GmbH, Göttingen, Germany). The resulting sequences were analyzed in Geneious Prime (Biomatters Inc.) and queried in GenBank and Barcode of Life (BOLD) databases (64, 65).

### Viral RNA extraction and PCR screening

RNA was extracted from sandfly homogenates using the Viral RNA Mini Kit (Qiagen, Hilden, Germany) according to the manufacturer’s protocol. A total of 10 µL of RNA was used to synthesize cDNA using the High Capacity cDNA Reverse Transcription Kit (Life Technologies, USA) and 600 μM non-ribosomal random hexanucleotide primers (66). A previously reported generic nested PCR targeting the phlebovirus RdRp gene was used to screen for viral RNA (67) using Platinum Taq DNA polymerase (Invitrogen GmbH, Karlsruhe, Germany). Bidirectional Sanger sequencing of all purified positive PCR products was performed by Microsynth Seqlab (Seqlab GmbH, Göttingen, Germany). To estimate the minimum phlebovirus infection rate in sandfly pools, data from phlebovirus detections were analyzed using PooledInfRate (https://www.cdc.gov/west-nile-virus/about/index.html) (68).

### Virus isolation

Samples positive for phlebovirus RNA were applied to virus isolation trials in sandfly (LL-5, *Lutzomyia longipalpis*; PP-9, *Phlebotomus papatasi*) and mammalian (Vero E6, African green monkey kidney) cell lines (69). Cells were inoculated with 50 μL of filtered and unfiltered sandfly homogenates in 48-well cell culture plates, incubated at 37°C with 5% CO_2_ for Vero E6 cells and at 28°C without CO_2_ for LL-5 and PP-9 cells, and examined daily for signs of CPE. Cell culture supernatants were tested for phleboviral genome copies using the RT-PCR described above.

### Sequence and phylogenetic analyses

Short nt sequences from conventional PCR and Sanger sequencing were analyzed in Geneious Prime (Biomatters Inc.) and queried against the GenBank nucleotide database in BLAST nucleotide searches (70).

FASTQ files from NGS were generated using a computational pipeline consisting of a SLURM Pipeline Python package (available at https://github.com/acorg/slurm-pipeline), and subsequently processed in Geneious Prime (Biomatters Inc.). The Geneious plugin BBDuk (https://www.geneious.com/plugins/bbduk) was used to remove low-quality reads and adapter sequences from paired-end reads. Sequencing reads were further analyzed without de-duplication to avoid removal of rare unique molecules, as well as with de-duplication where identical reads were removed. The resulting high-quality reads were mapped against suspected sandfly-borne phleboviruses or *de novo* assembled using the Geneious assembler in Geneious Prime (Biomatters Inc.) to obtain whole-genome sequences (65). Sequence gaps were filled by conventional PCR followed by Sanger sequencing (Microsynth Seqlab GmbH, Göttingen, Germany).

Obtained sequences were aligned with sequences of related viruses using the MAFFT E-INS-I plugin in Geneious (71). Maximum-likelihood phylogenetic analyses were performed with IQ-TREE 1.6.12 (72) using ModelFinder (73), ultrafast bootstrapping (74), and the following substitution models: GTR+F+I+G4 for the *COI* sandfly, RdRp, and GPC trees; TIM2e+I+G4 for the N tree; and TPM2u+F+I+G4 for the NSs tree. Nodal support was assessed by 1,000 bootstrap replicates (75).

## RESULTS

### Establishment of capture-based target enrichment NGS for sandfly-borne phleboviruses

**Fig 2:**
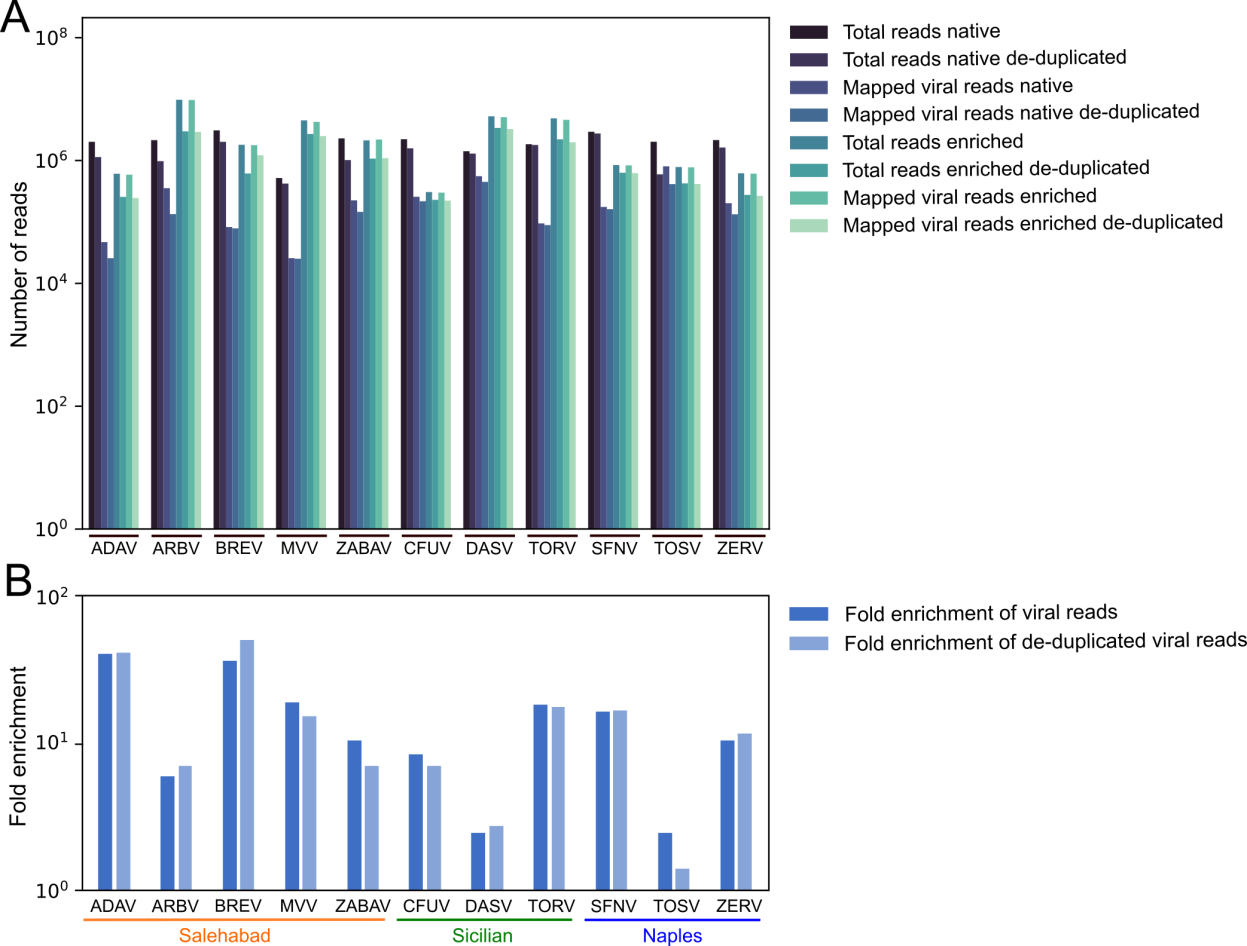
Phlebovirus genome sequencing read count from infectious cell culture supernatants. (**A**) Number of total generated reads and of mapped viral reads per sample is shown for native and capture-based target enrichment NGS. (**B**) Fold enrichment of viral reads per sample after capture.

**Fig 3:**
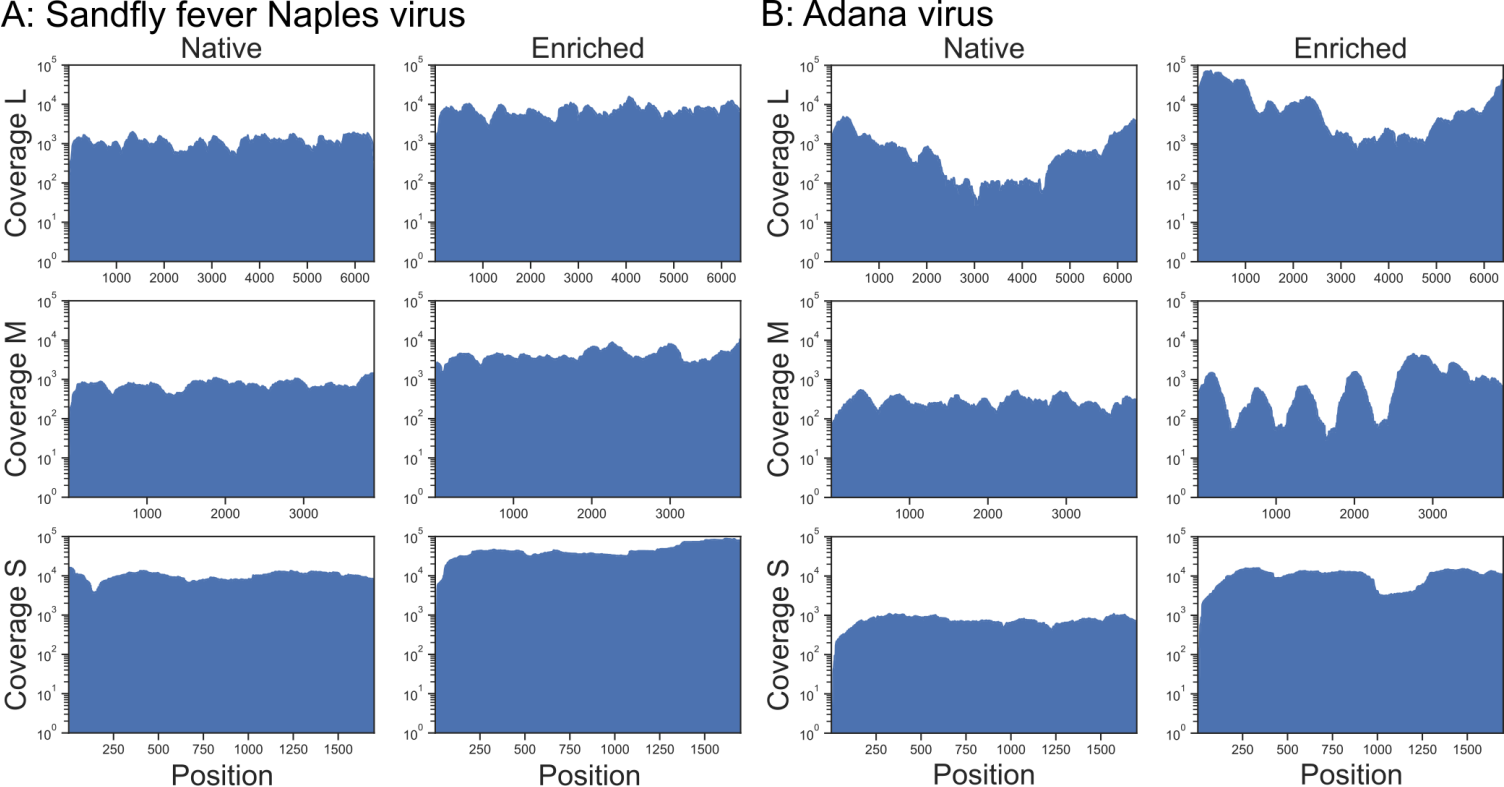
Phlebovirus genome sequencing results of native and capture-based target enrichment NGS from infectious cell culture supernatants. Genome coverage and coverage depth of L, M, and S segments per genome position without de-duplication are shown for (**A**) Sandfly fever Naples virus (sequence included in bait set design) and (**B**) Adana virus (sequence not included in bait set design). The *x*-axis position indicates the genomic location.

To evaluate the phlebovirus myBaits set, native and capture-based target enrichment NGS were performed using RNA from cell culture supernatants of 11 phlebovirus reference strains (Fig. 2). Native NGS generated between 498,712 and 2,961,360 total reads per sample (mean: 1,973,372). In enriched NGS, the number of total reads per sample was 295,344 to 9,300,700 (mean: 2,733,922) (Table 1). Total reads were reference-mapped to the L, M, and S segments of the respective virus sequences. Both approaches recovered whole-genome sequences for all 11 reference phleboviruses, as exemplified by SFNV, whose sequence was used as a reference for designing the baits, and by Adana virus (ADAV), whose sequence was not included in the bait set design (Fig. 3). Between 23,531 and 398,519 viral reads per million generated reads from native NGS were reference-mapped to the respective virus sequences, with a mean coverage ranging from 122 to 29,984.1 reads per genome position (Table 1; Tables S3 to S6). Reference mapping of enriched NGS reads to the respective virus sequences resulted in 946,638 to 1,030,379 viral reads per million generated reads, with a mean coverage ranging from 860.4 to 942,013.2 reads per genome position. To ensure that neither read count nor enrichment was artificially inflated, read counts before and after de-duplication were compared to each other, showing only minor differences, for example, de-duplication of the reads resulted in an up to 42-fold enrichment compared to a 41-fold enrichment of non-de-duplicated reads after capture (Fig. 2B).

**TABLE 1:** Sequencing statistics of native and capture-based target enrichment NGS for reference phleboviruses from infectious cell culture supernatants

Phlebovirus	No. of reads all segments							
	Native				Enriched			
	Total reads	Viral reads	Viral reads per million generated reads	Avg read length (bp)	Total reads	Viral reads	Viral reads per million generated reads	Avg read length (bp)
Adana virus	1,931,036	45,439	23,531	251	585,910	565,499	965,164	251
Arbia virus	2,055,998	341,275	165,990	251	9,300,700	9,228,328	992,219	251
Bregalaka virus	2,961,360	79,780	26,940	251	1,728,694	1,703,375	985,354	251
Corfou virus	2,128,378	247,414	116,245	251	295,344	289,098	978,852	251
Dashli virus	1,358,180	534,539	393,570	251	5,018,136	4,854,723	967,436	251
Medjerda Valley virus	498,712	24,916	49,961	251	4,305,838	4,076,810	946,810	251
Naples virus	2,812,964	169,160	60,136	251	810,786	802,087	989,271	251
Toros virus	1,765,918	91,498	51,813	251	4,640,672	4,393,036	946,638	251
Toscana virus	1,937,312	772,055	398,519	251	753,196	744,104	987,929	251
Zaba virus	2,195,528	216,445	98,584	251	2,038,656	2,100,589	1,030,379	251
Zerdali virus	2,061,704	194,457	94,319	251	595,214	587,660	987,309	251

### Sandfly collection and blood-meal source identification

**Fig 4:**
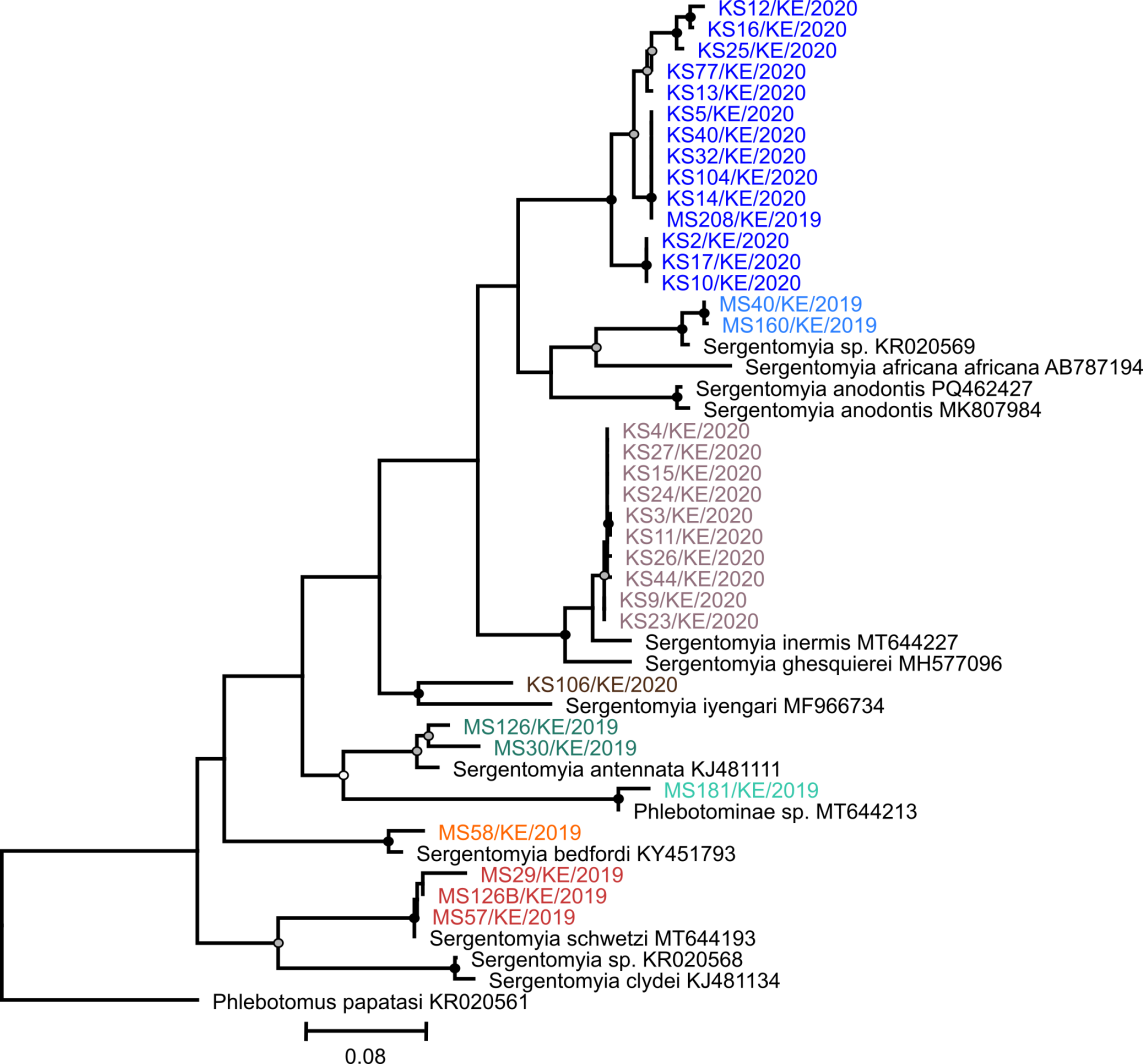
Phylogenetic analyses of partial *COI* sandfly sequences generated in this study. A maximum-likelihood phylogenetic analysis was based on a 528 nt MAFFT E-INS-i alignment, a GTR+F+I+G4 substitution model, and 1,000 bootstrap replicates. The tree was generated with IQ-TREE 1.6.12 (72) using ModelFinder (73) and ultrafast bootstrap (74). The tree was rooted to *Phlebotomus papatasi* KR020561. Bootstrap support values are represented by circles at the respective nodes, categorized as >95% (black), 76%–95% (gray), and 70%–75% (white).

A total of 15,652 sandflies were collected during the study period, 9,049 in Baringo County and 6,603 in Kajiado County (Table 2). Of these, 35 specimens were blood-fed and used for species identification based on DNA barcoding. A fragment of the mitochondrial *COI* gene was sequenced, revealing the presence of sandfly species grouping with *Sergentomyia inermis* (*n* = 10; 97% nt identity with *Se. inermis*), *Sergentomyia schwetzi* (*n* = 4; 97%–99% nt identity with *Se. schwetzi*), *Sergentomyia antennata* (*n* = 2; 95 and 97% nt identity with *Se. antennata*), *Sergentomyia bedfordi* (*n* = 1; 97% nt identity with *Se. bedfordi*), *Sergentomyia iyengari* (*n* = 1; 90% nt identity with *Se. iyengari*), a *Sergentomyia* sp. identified in Ethiopia (43) (*n* = 2; 98% nt identity with *Sergentomyia* sp. KR020569), and a *Phlebotominae* sp. identified in Kenya (*n* = 1; 98% nt identity with *Phlebotominae* sp. MT644213, [13]). Fourteen specimens were not closely related to specimens deposited in reference databases, forming a sister clade to the clade comprising *Sergentomyia anodontis, Sergentomyia africana africana,* and the *Sergentomyia* sp. identified in Ethiopia (Fig. 4).

**TABLE 2:** Collected sandflies from Baringo and Kajiado counties, Kenya

County	Sampling site	CDC-LT		BG
		Indoors	Outdoors	
Baringo	Kaptombes	0	1,059	0
	Kapkuikui	108	424	0
	Logumgum	56	423	0
	Sandai	3,884	1,324	0
	Ntepes	330	1,441	0
Kajiado	Olkirimatian	0	2,584	200
	Oloisinyai	0	1,269	0
	Soweto	0	1,747	803
Total		4,378	10,271	1,003
*n* of sandflies total		15,652		

Five vertebrate hosts were identified as blood meal host sources in 31 specimens, including humans (*Homo sapiens*), goats (*Capra hircus*), chickens (*Gallus gallus*), lizards (*Adolfus mathewsensis*), and tortoises (*Stigmochelys pardalis*). Humans were the most common host source (71.4%, 25/35). Mixed feeding on humans and chickens was found in one *Sergentomyia* sp. and one *Sergentomyia inermis* specimen. All blood-fed specimens were individually analyzed for the presence of phleboviruses, with all samples yielding negative results.

### Detection and genome sequencing of phleboviruses from primary sandfly material

The 15,652 specimens were pooled and tested for presence of phleboviruses. Ten of the pools tested (10/372; 2.69%) contained phlebovirus RNA. Short nt sequences of the RdRp gene of these viruses were identified as BGRV, EMBV, KBGV, and as two previously unknown phleboviruses. BGRV was detected in five sandfly pools (MS21/KE/2019, MS24/KE/2019, MS56/KE/2020, MS71/KE/2020, MS95/KE/2020) collected indoors and outdoors in Baringo County, EMBV was found in one sandfly pool (MS75/KE/2020) collected outdoors in Baringo County, and KBGV was detected in a sandfly pool (KS134/KE/2020) collected outdoors in Kajiado County. BGRV, EMBV, and KBGV were previously identified in Baringo County (7). The two potentially novel phlebovirus species were detected in three sandfly pools collected indoors and outdoors in Baringo County (MS134/KE/2020, MS141/KE/2020, and MS18/KE/2019). The short screening fragment of MS18/KE/2019 shared 99.78% maximum pairwise nt identity with a previously unknown virus detected in sandflies from Isiolo County, Kenya, in 2019 (SP109/KE/2019). The latter was included in this study for further analyses. Attempts to isolate the detected viruses in cell culture using LL-5, PP-9, and Vero E6 cells failed.

**Fig 5:**
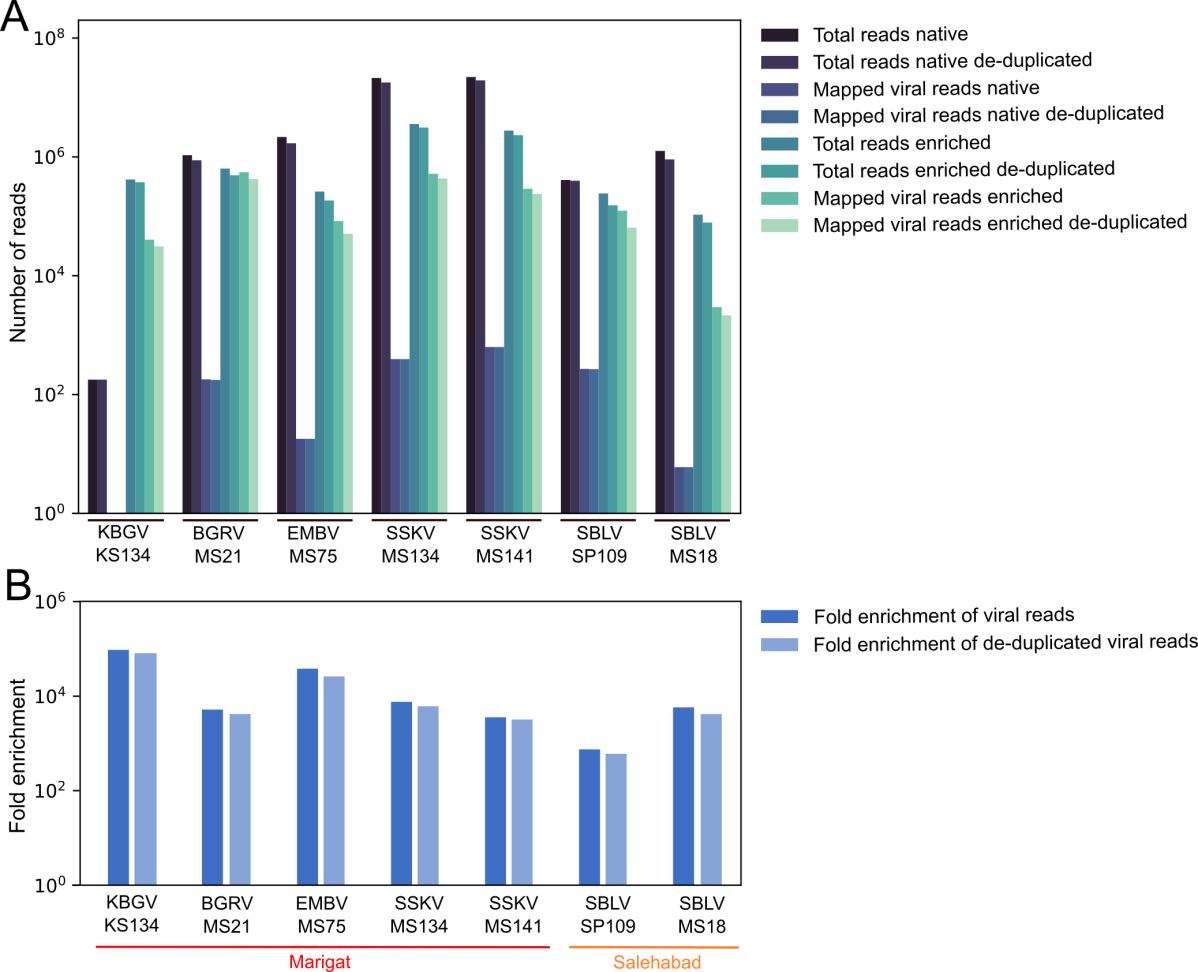
Phlebovirus genome sequencing read count from sandfly homogenates. (**A**) Number of total generated reads and of mapped viral reads per sample is shown for native and capture-based target enrichment NGS. (**B**) Fold enrichment of viral reads per sample after capture.

**Fig 6:**
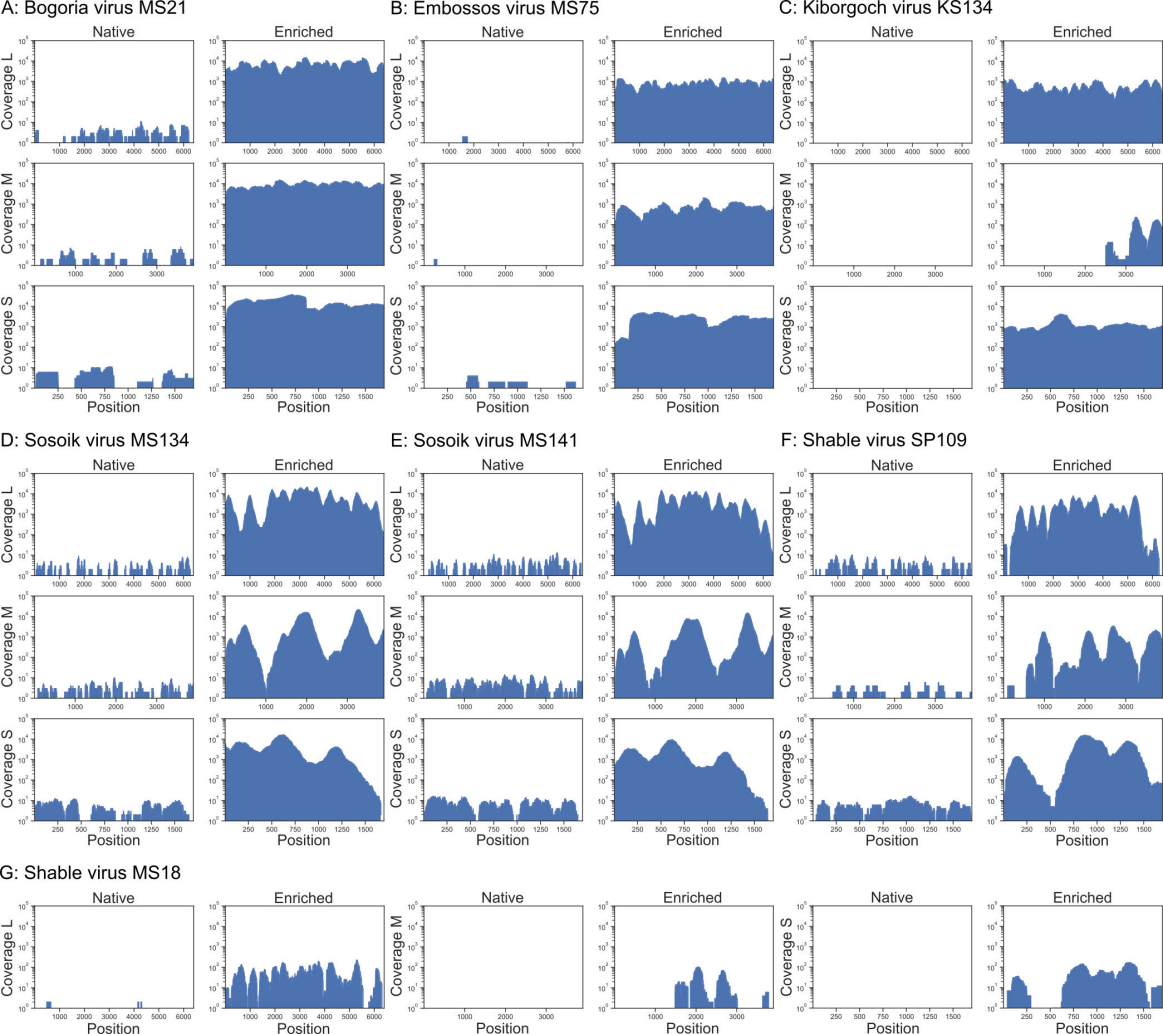
Phlebovirus genome sequencing results of native and capture-based target enrichment NGS from sandfly homogenates. Genome coverage and coverage depth of L, M, and S segments per genome position without de-duplication are shown for (**A**) BGRV MS21/KE/2019, (**B**) EMBV MS75/KE/2020, (**C**) KBGV KS134/KE/2020, (**D**) SSKV MS134/KE/2020, (**E**) SSKV MS141/KE/2020, (**F**) SBLV SP109/KE/2019, and (**G**) SBLV MS18/KE/2019. The *x*-axis position indicates the genomic location.

Seven PCR-positive sandfly homogenates were sequenced using native NGS and the newly established capture-based target enrichment NGS (Fig. 5). Native NGS generated between 178 and 22,097,992 total reads per sample (mean: 6,899,965). In enriched NGS, the number of total reads was reduced from 106,632 to 3,574,752 total reads per sample (mean: 1,144,721) (Table 3). Total reads were reference-mapped to the L, M, and S segments of related virus sequences. Native NGS yielded negative or poor-quality results with a low genome coverage for all samples (Fig. 6). In contrast, reference mapping of enriched NGS reads yielded near-complete genome coverage. Sequence gaps were closed using conventional PCR and full genomes were obtained for all viruses. Total reads were subsequently reference-mapped to their respective full genome sequences to detect any previously unmapped viral reads. However, this did only slightly improve the number of mapped viral reads for both approaches, resulting in 0 to 662 mapped viral reads per million generated reads from native NGS per sample, with a mean coverage ranging from 0 to 7.4 reads per genome position (Table 3; Tables S7 to S10). In contrast, reference mapping of enriched NGS reads to the respective full genome sequences resulted in 27,768 to 870,868 mapped viral reads per million generated reads, with a mean coverage ranging from 9.5 to 16,481.0 reads per genome position, indicating an up to 38,297-fold enrichment after capture (Fig. 5B). Comparison of read counts before and after de-duplication showed only minor deviations from each other indicating no significant inflation of non-de-duplicated reads (Fig. 5). There was an increase in the number of mapped viral reads from the native to the enriched libraries, ranging from 99.7% to 100% across the different segments. With regard to variation in bait set performance across genome segments, the M and S segments of some viruses showed slightly lower coverage depth compared to the L segment (Fig. S1). No additional sequence information was obtained for any of the samples by *de novo* assembly.

**TABLE 3:** Sequencing statistics of native and capture-based target enrichment NGS for seven phleboviruses originating from sandflies

Phlebovirus	No. of reads all segments							
	Native				Enriched			
	Total reads	Viral reads	Viral reads per million generated reads	Avg read length (bp)	Total reads	Viral reads	Viral reads per million generated reads	Avg read length (bp)
KBGV KS134/KE/2020	178	0	0	301	416,906	40,439	96,998	301
BGRV MS21/KE/2019	1,068,724	180	168	301	636,216	554,060	870,868	301
EMBV MS75/KE/2020	2,162,994	18	8	301	260,988	83,177	318,700	301
SSKV MS134/KE/2020	21,299,276	395	19	120	3,574,752	519,574	145,345	144
SSKV MS141/KE/2020	22,097,992	631	29	131	2,774,922	291,089	104,900	141
SBLV SP109/KE/2019	408,130	270	662	301	242,628	124,204	511,911	301
SBLV MS18/KE/2019	1,262,464	6	5	301	106,632	2,961	27,768	301

#### Detection of previously known sandfly-borne phleboviruses in the Marigat virus serocomplex

Capture-based target enrichment NGS recovered complete coding sequences for variants of BGRV (MS21/KE/2019) and EMBV (MS75/KE/2020) (Fig. 6). The BGRV MS21/KE/2019 CDS shared maximum pairwise nt identities with other BGRV strains from Baringo of 88.66 to 99.60% in the RdRp, 96.28 to 98.05% in the GPC, 98.58 to 98.97% in the NSs, and 98.24 to 99.86% in the N genes. The EMBV MS75/KE/2020 CDS shared maximum pairwise nt identities with the EMBV strain from 2016 of 99.46% in the RdRp, 99.3% in the GPC, 99.23% in the NSs, and 99.86% in the N genes. Partial coding genome sequences were recovered for KS134/KE/2020, corresponding to KBGV (Fig. 6). Sequence gaps were filled by conventional PCR, resulting in the recovery of the entire CDS sequences. KBGV KS134/KE/2020 CDS shared maximum pairwise nt and amino acid identities (aa) of 89.93% and 98.09% with the KBGV strain from 2016 in the RdRp, 65.43% and 67.92% in the GPC, 85.61% and 89.58% in the NSs, and 91.47% and 100% in the N genes.

#### Detection of two previously unknown phleboviruses in the Salehabad and Marigat serocomplexes

Partial coding genome sequences were recovered for MS134/KE/2020, MS141/KE/2020, MS18/KE/2019, and SP109/KE/2019 (Fig. 6). Sequence gaps were filled by conventional PCR, resulting in coding-complete sequences for all viruses. MS134/KE/2020 and MS141/KE/2020 were two strains of a potentially novel virus species closely related to BGRV, tentatively named Sosoik virus (SSKV). In addition, two strains of a second potentially novel virus species in the Salehabad serocomplex, tentatively named Shable virus (SBLV), were detected (MS18/KE/2019, SP109/KE/2019). Names for the new viruses are based on the local names for sandfly in the counties where the virus-positive sandflies were collected (Baringo and Isiolo counties, respectively).

The coding-complete sequences of SBLV and SSKV viruses showed the characteristic phlebovirus genome organization, consisting of a tripartite genome with an L segment encoding the RdRp, an M segment encoding the two glycoproteins Gc and Gn, and NSm, and an S segment encoding NSs and N (Table 4).

**TABLE 4:** Phleboviruses detected in Baringo and Kajiado counties, Kenya

Virus	Strain	County	Sampling site	Accession no.
Bogoria virus	BGRV MS24/KE/2019	Baringo	Ntepes	L: PV963140
	BGRV MS56/KE/2020	Baringo	Kaptombes	L: PV963141
	BGRV MS71/KE/2020	Baringo	Kaptombes	L: PV963142
	BGRV MS95/KE/2020	Baringo	Sandai	L: PV963143
	BGRV MS21/KE/2019	Baringo	Logumgum	L: PV963144
				M: PV963138
				S: PV963139
Embossos virus	EMBV MS75/KE/2020	Baringo	Kapkuikui	L: PV963145
				M: PV963146
				S: PV963147
Kiborgoch virus	KBGV KS134/KE/2020	Kajiado	Oloisinyai	L: PV963148
				M: PV963149
				S: PV963150
Shable virus	SBLV SP109/KE/2019	Isiolo	Malka Galla	L: PV963161
				M: PV963159
				S: PV963157
	SBLV MS18/KE/2019	Baringo	Ntepes	L: PV963162
				M: PV963160
				S: PV963158
Sosoik virus	SSKV MS134/KE/2020	Baringo	Sandai	L: PV963155
				M: PV963153
				S: PV963151
	SSKV MS141/KE/2020	Baringo	Logumgum	L: PV963156
				M: PV963154
				S: PV963152

SBLV MS18/KE/2019 was found to be a reassortant virus sharing its L and S segments with SBLV SP109/KE/2019 and the M segment with an undescribed Ponticelli-like virus. Pairwise nt and aa identities of their RdRp genes were 99.03% and 99.86%, respectively, and 98.78% and 99.63% in their NSs, and 98.6% and 100% in their N genes. The M segment of SBLV MS18/KE/2019 shared 71.64 to 71.97% and 79.82 to 80.12% pairwise nt and aa identities with Ponticelli I viruses, respectively. The aa sequences of the RdRp of both SBLV strains showed maximum pairwise identities of 88% to the RdRp genes of other members of the Salehabad serocomplex. According to the ICTV species demarcation criteria, SBLV SP109/KE/2019 and SBLV MS18/KE/2019 are two strains of a novel species in the Salehabad serocomplex, with SBLV MS18/KE/2019 being a reassortant virus.

The CDS sequences of SSKV MS134/KE/2020 and SSKV MS141/KE/2020 showed nt and aa pairwise identities of 99.28% and 99.86% in the RdRp, 99.3% and 99.55% in the GPC, 99.61% and 100% in the NSs, and 99.46% and 100% in their N genes. The aa sequences of both SSKV strains showed maximum pairwise identities of 83% in their RdRp proteins to those of BGRV. According to the ICTV species demarcation criteria, SSKV MS134/KE/2020 and SSKV MS141/KE/2020 are two strains of a novel species.

### Phylogenetic relationship of detected phleboviruses

**Fig 7:**
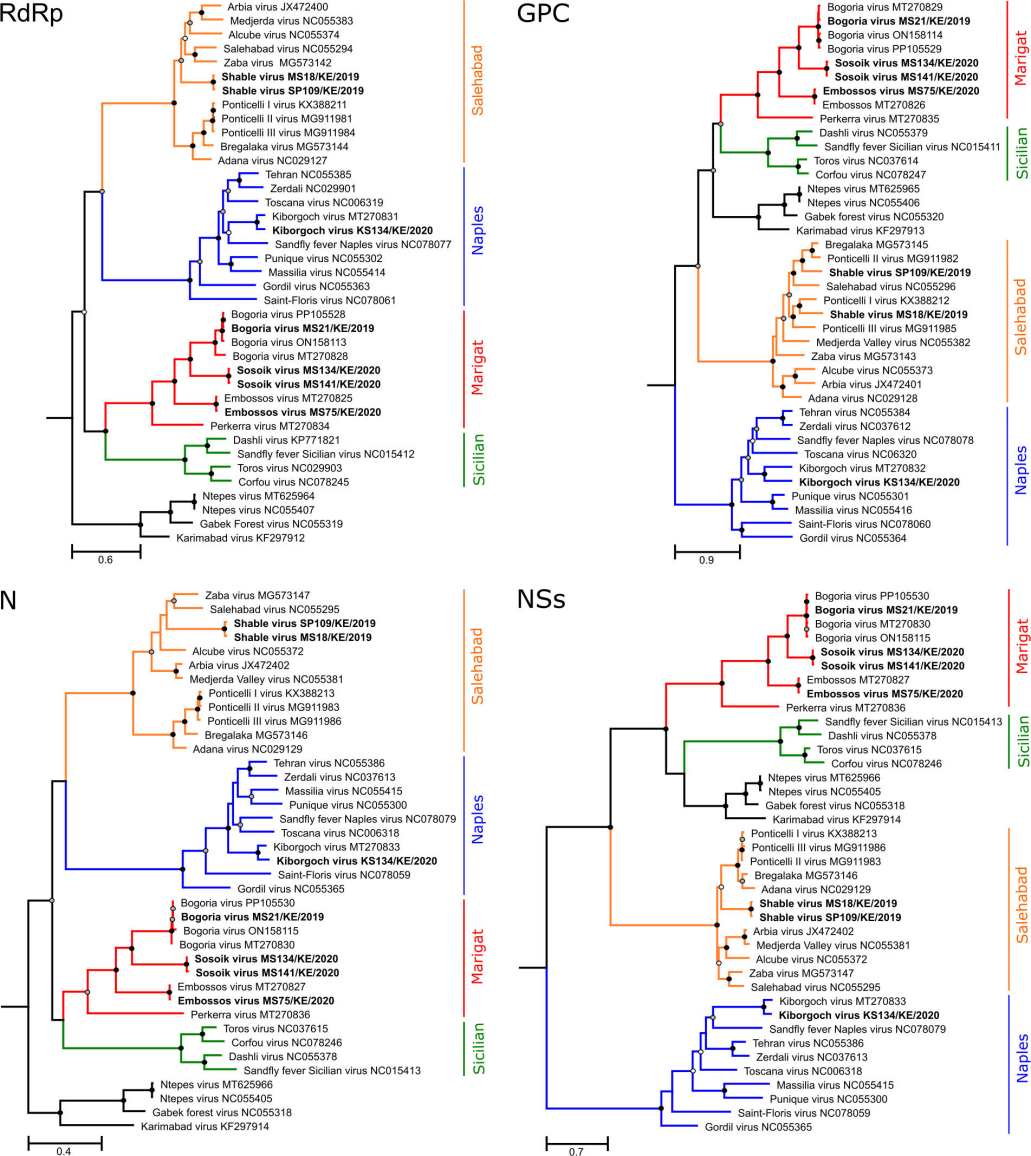
Phylogenetic analyses of newly identified sandfly-borne phleboviruses. Maximum-likelihood phylogenetic analyses were based on alignments of 5,970 nt of the RdRp gene, 2,449 nt of the GPC gene, 744 nt of the N gene, and 1,052 nt of the NSs gene (MAFFT E-INS-i protein alignment, GTR+F+I+G4 [RdRp and GPC], TIM2e+I+G4 [N] and TPM2u+F+I+G4 [NSs] substitution models, 1,000 bootstrap replicates). Trees were generated with IQ-TREE 1.6.12 (72) using ModelFinder (73) and ultrafast bootstrap (74). All trees were rooted to Mukawa virus NC043510 (RdRp), NC043509 (GPC), NC043511 (N), and NC043511 (NSs). Viruses identified in this study are shown in bold. Orange, Salehabad serocomplex; green, Sicilian serocomplex; red, Marigat serocomplex; blue, Naples serocomplex. Bootstrap support values are represented by circles at the respective nodes, categorized as >95% (black), 76%–95% (gray), and 70%–75% (white).

To infer the evolutionary relationship of the detected viruses, genome sequences of the major genes of all ICTV-classified sandfly-borne phlebovirus species were aligned, and phylogenetic trees were inferred. Phylogenetic analyses based on the RdRp, GPC, N, and NSs genes showed that the strains BGRV MS21/KE/2019, EMBV MS75/KE/2020, and KBGV KS134/KE/2020 consistently clustered with their respective reference viruses, BGRV, EMBV, and KBGV, across all trees (Fig. 7). Similarly, both SSKV strains consistently formed a sister group to BOGV in all phylogenies. In contrast, the two SBLV strains (SP109/KE/2019 and SBLV MS18/KE/2019) showed evidence of reassortment. In phylogenetic trees based on the RdRp, N, and NSs genes, both SBLV strains grouped together on a long branch within the Salehabad serocomplex, indicating a shared evolutionary origin. However, in the GPC gene-based tree (representing the M segment), the strains showed divergent phylogenetic relationships. The M segment of SBLV SP109/KE/2019 was closely related to Ponticelli II and Bregalaka viruses, whereas the M segment of SBLV MS18/KE/2019 was placed as a sister taxon to Ponticelli I virus.

## DISCUSSION

Primary isolation of phleboviruses in cell culture can be difficult, and full genome sequencing from primary material remains a challenge (7, 9, 38, 41). Here, we describe a capture-based target enrichment NGS approach for sandfly-borne phlebovirus genome sequencing as a robust tool that can retrieve full genome information of sandfly-borne phleboviruses from primary material of all medically important serogroups and detect previously unknown phleboviruses with nt divergence of up to 29%. Notably, native NGS yielded negative or poor-quality results for all samples, while up to 38,297-fold enrichment from primary material after capture was achieved.

The bait set was successfully tested on RNA from infectious cell culture supernatants of 11 reference sandfly-borne phleboviruses and enabled the coding-complete genome sequencing of BGRV, EMBV, KBGV, and of two previously unknown viruses (SSKV and SBLV) from sandfly primary material. Several studies have already demonstrated the usability of capture-based target enrichment NGS for the recovery of entire genomes from primary material (38, 41, 76–83), including arthropod samples (79, 84–87).

Our study presents a genus-specific bait set designed for the targeted enrichment of sandfly-borne phleboviruses, addressing a gap left by existing universal bait sets that underrepresent these viruses. By focusing on a specific vector group and its associated viruses, our approach offers several advantages, including efficient detection and full genome recovery of both known and novel phleboviruses, supported by improved sensitivity and higher probe density per target. Universal enrichment methods provide broad-spectrum viral detection across hundreds of viral taxa but are often limited by low probe density for individual species (76). This limitation is particularly relevant for segmented viruses like phleboviruses, where the highly variable M segment complicates complete genome assembly. Additionally, universal approaches are expensive and may not be cost-effective for routine surveillance or research focused on specific virus groups. In clinical diagnostics, PCR-based methods remain the preferred first-line tests due to their speed, cost-efficiency, and simplicity. However, targeted NGS with genus-specific bait sets serves as a valuable complementary tool for confirming PCR-positive cases and achieving full genome characterization, which is critical for understanding viral diversity, evolution, and geographic distribution. This is especially important in regions with high sandfly prevalence and limited diagnostic resources, such as parts of East Africa, where fever of unknown origin outbreaks frequently occur. As sequencing technologies become more accessible and affordable, genus-specific enrichment strategies may become a practical tool not only for research but also for enhanced surveillance and clinical investigation in endemic settings. By improving our understanding of the geographic distribution and genetic diversity of phleboviruses, these approaches can guide the development of improved diagnostics and targeted disease management strategies.

Uneven coverage observed in the M segment for certain viruses, such as Adana virus, appears to be sample-specific rather than a general limitation of the bait set. This may result from localized sequence variability or absence of exact sequence representation in the bait design. Nevertheless, the bait set is designed to tolerate high sequence divergence across the genus *Phlebovirus* and demonstrates consistent performance across most samples. While local sequence variability may occasionally influence capture efficiency, the overall performance across diverse phleboviruses indicates robust capture despite genomic divergence.

The present study focuses on developing a capture-based target enrichment method optimized for low-input material, where retaining all reads, including potential duplicates, is essential to avoid underestimating enrichment efficiency and sensitivity. Although de-duplication is commonly used to remove PCR duplicates in high-complexity samples and is crucial for analyses requiring accurate quantification of original molecules, such as gene expression and variant calling, it can lead to the removal of genuinely unique molecules in low-input samples with inherently limited complexity. To accurately assess the performance of the capture, the percentage of viral reads before and after enrichment, both with and without de-duplication, was compared, indicating no evidence for an artificial inflation of non-de-duplicated reads. In the context of this study, de-duplication provided no benefit. Instead, it resulted in the loss of a small number of unique sequence reads that were necessary to achieve complete genome coverage.

Novel sandfly-borne phleboviruses, including NTPV, EMBV, BGRV, PERV, and KBGV, have been discovered during arbovirus surveillance studies conducted in Baringo County, Kenya, from 2014 to 2016 (6, 7). In the present study, further evidence for a wider distribution and greater genetic diversity of sandfly-borne phleboviruses in Kenya was provided. Here, known and previously unknown phleboviruses were detected in sandflies from Baringo, Kajiado, and Isiolo counties. BGRV and EMBV were detected again in sandflies from Baringo County, where they were first detected in 2016 (7). KBGV, previously identified in sandflies from Baringo County in 2016 (7), was now found in sandflies from Kajiado County. EMBV, BGRV, and KBGV were circulating 4 years after their first detection and were detected in other parts of the country as well. The findings in the present study provide evidence that sandfly-borne phleboviruses of all medically important serocomplexes co-circulate in Kenya. Co-circulation of sandfly-borne phleboviruses has also been observed in other studies, such as Toros, ADAV, and Zerdali viruses in Turkey (12), TOSV, Corfou, Fermo, and Ponticelli viruses in Italy (16), and Alcube and Massilia viruses in Portugal (88). Co-circulation can lead to simultaneous infection of an individual with multiple viruses and may lead to reassortant progeny. Infections of an insect vector or vertebrate host with multiple viruses are the underlying conditions for segment reassortment, consisting mainly of an exchange of the M segment between phleboviruses and are an important driver of virus evolution (89). Reassortant phleboviruses are, for example, Aguacate virus from Panama (90), Granada virus from Spain (91), and Arrabida virus from Portugal (92).

Here, in addition to the detection of known phleboviruses, two potentially novel phlebovirus species, including one reassortant virus, were identified and tentatively named SSKV (strains MS134/KE/2020 and MS141/KE/2020), and SBLV (strains Sp109/KE/2019 and MS18/KE/2019). The latter shared the L and S segments with SBLV and the M segment with an undescribed Ponticelli-like virus. SSKV established a sister taxon to BGRV, with a maximum pairwise aa identity of 83% in its RdRp gene. The two SBLV viruses showed a maximum pairwise aa identity of 88% in their RdRp genes with viruses of the Salehabad serocomplex and are most likely a member of that serocomplex. These data suggest that SSKV and SBLV viruses are two novel phlebovirus species, according to the ICTV phlebovirus species demarcation criteria, which define a new species based on an aa sequence divergence of greater than 5% in the RdRp from the closest related virus (93). Other viruses recognized by the ICTV in the Salehabad serocomplex include Medjerda Valley virus (*Phlebovirus medjerdaense*) and Adana virus (*Phlebovirus adanaense*, ADAV) isolated in Tunisia and Turkey, respectively (10, 13). Species in the Salehabad serocomplex have historically been considered to be of limited veterinary and public health importance (13, 94). However, the report of a human infection with febrile illness caused by Adria virus in Greece, and high ADAV seroprevalence rates in domestic animals in Turkey (10, 33) suggest that viruses of the Salehabad serocomplex may have public health implications. This is the first detection of a virus from this serocomplex in Kenya and further expands the genetic diversity of sandfly-borne phleboviruses in the country (6, 7).

Simultaneously with virus detection, bionomic traits of sandflies, such as blood-feeding pattern and species identity, were analyzed by *COI* barcoding PCR (63). The blood-fed sandfly specimens belonged primarily to the genus *Sergentomyia* (e.g.*, S. inermis* and *S. schwetzi*), which have been implicated as likely vectors of NTPV (6). Other identified sandfly species included *S. antennata* and *S. bedfordi*. This is consistent with *Sergentomyia* sandfly species previously identified in the Kenyan fauna (95–97). Sequence analysis of 14 sandfly specimens suggests the detection of four potentially undescribed species, highlighting the need for further entomological surveys to describe sandfly diversity and their potential role in virus transmission. Future studies of sandfly species diversity should employ combined molecular analysis approaches using *COI* and other markers such as cytochrome B, NADH dehydrogenase 4, and internal transcribed spacer to overcome the limitations of using only one marker and to provide a more comprehensive phylogeny (98).

Human blood was detected in sandflies collected both inside and outside human dwellings, indicating human exposure to sandfly bites and associated pathogens they may carry. The previously undescribed sandfly species collected in this study also fed on humans, suggesting that they may play a role in phlebovirus circulation in the study areas. There was no evidence of sandflies feeding on rodents, which are considered a potential reservoir for sandfly-borne phleboviruses (6, 99). Rodents are abundant in the sampled localities, but only a few blood-fed sandflies were analyzed, limiting inferences about feeding patterns. To date, data on potential amplifying or reservoir hosts of the detected phleboviruses are unknown. Studies of virus maintenance and transmission under laboratory and natural conditions are warranted.

### Conclusion

We have successfully established a capture-based NGS approach for sandfly-borne phleboviruses. We demonstrated that it is a suitable and robust tool to generate complete genomes of known phleboviruses and entirely new species from all human-pathogenic serogroups. We recovered genomes of 14 previously known phleboviruses, including 11 reference strains sequenced from infectious cell culture supernatants and seven variants belonging to three phleboviruses previously identified in Kenya, Embossos, Bogoria, and Kiborgoch viruses. In addition, we detected two previously unknown phlebovirus species, which we named Sosoik and Shable viruses. Two variants of the Shable virus were identified, one of which represents a reassortant. Our findings provide evidence for the circulation and endemicity of sandfly-borne phleboviruses in Kenya and demonstrate that targeted enrichment NGS significantly improves the recovery of whole phlebovirus genomes from sandfly primary material. In addition, the study discovered two novel sandfly-borne phlebovirus species, tentatively named SBLV and SSKV. SSKV is related to BGRV, which is endemic in Kenya. SBLV is related to viruses of the Salehabad serocomplex. This is the first detection of viruses of this serocomplex in Kenya. Our long-term studies contributed significantly to the understanding of the geographical distribution of sandfly-borne phleboviruses and their genetic diversity by providing complete genome information of seven previously unknown sandfly-borne phleboviruses (BGRV, EMBV, KBGV, NTPV, PERV, SBLV, SSKV) to the previously classified 18 Old-World sandfly-borne phlebovirus species, an increase of 38.9%. Overall, we have identified seven novel species in Kenya from all known sandfly-borne phlebovirus serocomplexes and described a new serocomplex, the Marigat serocomplex (7). The close relationship of the novel viruses to sandfly fever viruses highlights the need to investigate the potential contribution of the novel phleboviruses to fevers of unknown origin in the study sites and beyond, in areas where the vector is present. Epidemiologic studies to assess the amplification, transmission, and pathogenicity of the detected sandfly-borne phleboviruses in humans, livestock, and wildlife are recommended.

## Data Availability

The sequences of this study were deposited in GenBank under the accession numbers PV963138 to PV963162. The reads of the capture-based enrichment NGS were deposited in the Sequence Read Archive (SRA) database under the BioProject accession number PRJNA1367297 and under the BioSample accession numbers SAMN53336877 to SAMN53336894.

## SUPPLEMENTAL MATERIAL

Tables S1 to S10; Figure S1 jcm.01082-25-s0001.docx Sequence statistics and sample information.

ASM does not own the copyrights to Supplemental Material that may be linked to, or accessed through, an article. The authors have granted ASM a non-exclusive, world-wide license to publish the Supplemental Material files. Please contact the corresponding author directly for reuse.
