# Supplementary material for: Identification and full genome sequencing of previously unknown sandfly-borne phleboviruses using a newly established capture-based next-generation sequencing approach
Source: J Clin Microbiol. 2026 Mar 13;64(4):e01082-25. doi: 10.1128/jcm.01082-25 (PMC13059712; doi:10.1128/jcm.01082-25)
Supplement: Tables S1 to S10; Figure S1 — Sequence statistics and sample information. [file jcm.01082-25-s0001.docx]

**Supplementary Material**

**“Identification and full genome sequencing of previously unknown sandfly-borne phleboviruses using a newly established capture-based next generation sequencing approach”**

**Table S1:** Sequences used for the design of the myBaits® set.

| **Virus name** | **Strain** | **Accession numbers** | | |
| --- | --- | --- | --- | --- |
|  |  | **L-segment** | **M-segment** | **S-segment** |
| Bogoria virus | SP105-KE-2016 | MT270828 | MT270829 | MT270830 |
| Bregalaka virus | M31 | MG573144 | MG573145 | MG573146 |
| Corfou virus | PaAr814 | KR106177 | KR106178 | KR106179 |
| Dashli virus | 131 | KP771821 | KP771822 | KP771823 |
| Embossos virus | SP288-KE-2016 | MT270825 | MT270826 | MT270827 |
| Kiborgoch virus | SSP39-KE-2016 | MT270831 | MT270832 | MT270833 |
| Medjerda Valley virus | T131 | NC055383 | NC055382 | NC055381 |
| Naples virus | Yu8/76 | JF920139 | JF920140 | JF920141 |
| Perkerra virus | SP166-KE-2016 | MT270834 | MT270835 | MT270836 |
| Sand fever Naples-like virus | - | HM566183 | HM566184 | HM566182 |
| Sand fever Naples-like virus | Poona | HM566176 | HM566177 | HM566178 |
| Sandfly fever Naples virus | - | HM566172 | HM566171 | HM566170 |
| Sandfly fever Sicilian virus | Izmir | NC015412 | NC015411 | NC015413 |
| Tehran virus | I-47 | JF939846 | JF939847 | JF939848 |
| Toros virus | 213 | NC029903 | NC037614 | NC037615 |
| Toscana virus | Toscana | NC006319 | NC006320 | NC006318 |
| Toscana virus | A189 | KP694240 | KP694241 | KP694242 |
| Toscana virus | Nice/113 | KU204983 | KU204982 | KU204981 |
| Zerdali virus | 37 | NC_029901 | NC_037612 | NC_037613 |

**Table S2:** Virus isolates of reference viruses used for the evaluation of the myBaits® set.

| **Virus name** | **Abbreviation** | **Strain** |
| --- | --- | --- |
| Adana virus | ADAV | UVE/ADAV/2012/TR/195 |
| Arbia virus | ARBV | UVE/ARBV/UNK/ITPhl.35 M6 |
| Bregalaka virus | BREV | UVE/BREV/2015/MK/M31 |
| Corfou virus | CFUV | UVE/CFUV/UNK/GR/PaAr 814 |
| Dashli virus | DASV | UVE/DASV/2011/IR/90 |
| Medjerda Valley virus | MVV | UVE/MVV/2010/TN/T131 |
| Sandfly fever Naples virus | SFNV | UVE/SFNV/UNK/IT/30451 |
| Toros virus | TORV | UVE/TORV/2012/TR/292 |
| Toscana virus | TOSV | UVE/TOSV/2004/FR/H4906 |
| Zaba virus | ZABAV | UVE/ZABAV/2015/HR/C48 |
| Zerdali virus | ZERV | UVE/ZERV/2013/TR/37 |

**Table S3:** Sequencing statistics of native and capture-based target enrichment NGS for the L-segment of reference phleboviruses from infectious cell culture supernatants.

| **Phlebovirus** | **No. of reads L-segment** | | | | | |
| --- | --- | --- | --- | --- | --- | --- |
|  | **Native** | | | **Enriched** | | |
|  | **Total reads** | **Viral reads** | **Viral reads per million generated reads** | **Total reads** | **Viral reads** | **Viral reads per million generated reads** |
| Adana virus | 1,931,036 | 31,784 | 16,460 | 585,910 | 461,593 | 787,822 |
| Arbia virus | 2,055,998 | 49,752 | 24,198 | 9,300,700 | 965,241 | 103,782 |
| Bregalaka virus | 2,961,360 | 28,691 | 9,688 | 1,728,694 | 591,041 | 341,900 |
| Corfou virus | 2,128,378 | 52,247 | 24,548 | 295,344 | 65,496 | 221,762 |
| Dashli virus | 1,358,180 | 183,987 | 135,466 | 5,018,136 | 1,764,951 | 351,714 |
| Medjerda Valley virus | 498,712 | 19,416 | 38,932 | 4,305,838 | 3,063,186 | 711,403 |
| Naples virus | 2,812,964 | 40,317 | 14,333 | 810,786 | 239,819 | 295,786 |
| Toros virus | 1,765,918 | 34,722 | 19,662 | 4,640,672 | 2,081,167 | 448,462 |
| Toscana virus | 1,937,312 | 222,515 | 114,858 | 753,196 | 260,030 | 345,236 |
| Zaba virus | 2,195,528 | 80,092 | 36,480 | 2,038,656 | 900,977 | 441,947 |
| Zerdali virus | 2,061,704 | 49,818 | 24,164 | 595,214 | 177,599 | 298,378 |

**Table S4:** Sequencing statistics of native and capture-based target enrichment NGS for the M-segment of reference phleboviruses from infectious cell culture supernatants.

| **Phlebovirus** | **No. of reads M-segment** | | | | | |
| --- | --- | --- | --- | --- | --- | --- |
|  | **Native** | | | **Enriched** | | |
|  | **Total reads** | **Viral reads** | **Viral reads per million generated reads** | **Total reads** | **Viral reads** | **Viral reads per million generated reads** |
| Adana virus | 1,931,036 | 6,156 | 3,188 | 585,910 | 18,307 | 31,245 |
| Arbia virus | 2,055,998 | 46,856 | 22,790 | 9,300,700 | 167,462 | 18,005 |
| Bregalaka virus | 2,961,360 | 45,055 | 15,214 | 1,728,694 | 993,744 | 574,852 |
| Corfou virus | 2,128,378 | 18,852 | 8,857 | 295,344 | 23,362 | 79,101 |
| Dashli virus | 1,358,180 | 32,544 | 23,961 | 5,018,136 | 341,925 | 68,138 |
| Medjerda Valley virus | 498,712 | 2,668 | 5,350 | 4,305,838 | 525,965 | 122,152 |
| Naples virus | 2,812,964 | 17,535 | 6,234 | 810,786 | 108,623 | 133,972 |
| Toros virus | 1,765,918 | 15,824 | 8,961 | 4,640,672 | 684,261 | 147,449 |
| Toscana virus | 1,937,312 | 142,562 | 73,588 | 753,196 | 165,487 | 219,713 |
| Zaba virus | 2,195,528 | 47,372 | 21,577 | 2,038,656 | 501,368 | 245,931 |
| Zerdali virus | 2,061,704 | 40,653 | 19,718 | 595,214 | 140,949 | 236,804 |

**Table S5:** Sequencing statistics of native and capture-based target enrichment NGS for the S-segment of reference phleboviruses from infectious cell culture supernatants.

| **Phlebovirus** | **No. of reads S-segment** | | | | | |
| --- | --- | --- | --- | --- | --- | --- |
|  | **Native** | | | **Enriched** | | |
|  | **Total reads** | **Viral reads** | **Viral reads per million generated reads** | **Total reads** | **Viral reads** | **Viral reads per million generated reads** |
| Adana virus | 1,931,036 | 7,499 | 3,883 | 585,910 | 85,599 | 146,096 |
| Arbia virus | 2,055,998 | 244,667 | 119,002 | 9,300,700 | 8,095,625 | 870,432 |
| Bregalaka virus | 2,961,360 | 6,034 | 2,038 | 1,728,694 | 118,590 | 68,601 |
| Corfou virus | 2,128,378 | 176,315 | 82,840 | 295,344 | 200,240 | 677,989 |
| Dashli virus | 1,358,180 | 318,008 | 234,143 | 5,018,136 | 2,747,847 | 547,583 |
| Medjerda Valley virus | 498,712 | 2,832 | 5,679 | 4,305,838 | 487,659 | 113,255 |
| Naples virus | 2,812,964 | 111,308 | 39,570 | 810,786 | 453,645 | 559,513 |
| Toros virus | 1,765,918 | 40,952 | 23,190 | 4,640,672 | 1,627,608 | 350,727 |
| Toscana virus | 1,937,312 | 406,978 | 210,074 | 753,196 | 318,587 | 422,980 |
| Zaba virus | 2,195,528 | 88,981 | 40,528 | 2,038,656 | 698,244 | 342,502 |
| Zerdali virus | 2,061,704 | 103,986 | 50,437 | 595,214 | 269,112 | 452,126 |

**Table S6:** Average sequencing depth per segment of native and capture-based target enrichment NGS for reference phleboviruses from infectious cell culture supernatants.

| **Phlebovirus** | **Average sequencing depth** | | | | | |
| --- | --- | --- | --- | --- | --- | --- |
|  | **L** | | **M** | | **S** | |
|  | **native** | **enriched** | **native** | **enriched** | **native** | **enriched** |
| Adana virus | 911.7 | 17,426.7 | 276.4 | 953.3 | 726.5 | 9,939.2 |
| Arbia virus | 1,442.5 | 32,571.4 | 2,054.3 | 9,025.0 | 24,708.0 | 942,013.2 |
| Bregalaka virus | 867.7 | 18,636.9 | 2,113.6 | 45,360.2 | 654.4 | 12,292.7 |
| Corfou virus | 1,422.8 | 1,677.2 | 745.5 | 860.4 | 16,002.7 | 17,448.7 |
| Dashli virus | 5,434.0 | 52,863.5 | 1,407.4 | 14,438.1 | 29,984.1 | 245,207.0 |
| Medjerda Valley virus | 598.5 | 110,987.8 | 122.0 | 23,683.8 | 266.9 | 46,702.5 |
| Naples virus | 1,158.3 | 7,046.7 | 754.1 | 4,739.5 | 9,317.5 | 46,294.0 |
| Toros virus | 1,077.4 | 66,391.2 | 708.1 | 32,505.5 | 4,611.3 | 203,223.3 |
| Toscana virus | 5,718.5 | 7,509.0 | 5,252.4 | 7,289.7 | 25,298.7 | 36,615.1 |
| Zaba virus | 2,233.6 | 36,145.0 | 1,988.4 | 24,347.7 | 8,563.7 | 88,137.6 |
| Zerdali virus | 1,208.0 | 5,051.4 | 1,476.9 | 5,890.0 | 8,707.1 | 24,430.5 |

**Table S7:** Sequencing statistics of native and capture-based target enrichment NGS for the L-segment of seven phleboviruses originating from sandfly samples.

| **Phlebovirus** | **No. of reads L-segment** | | | | | |
| --- | --- | --- | --- | --- | --- | --- |
|  | **Native** | | | **Enriched** | | |
|  | **Total reads** | **Viral reads** | **Viral reads per million generated reads** | **Total reads** | **Viral reads** | **Viral reads per million generated reads** |
| KBGV KS134/KE/2020 | 178 | 0 | 0 | 416,906 | 23,492 | 56,348 |
| BGRV MS21/KE/2019 | 1,068,724 | 88 | 82 | 636,216 | 217,128 | 341,280 |
| EMBV MS75/KE/2020 | 2,162,994 | 4 | 2 | 260,988 | 33,385 | 127,918 |
| SSKV MS134/KE/2020 | 21,299,276 | 145 | 7 | 3,574,752 | 352,286 | 98,548 |
| SSKV MS141/KE/2020 | 22,097,992 | 222 | 10 | 2,774,922 | 205,147 | 73,929 |
| SBLV SP109/KE/2019 | 408,130 | 130 | 319 | 242,628 | 76,908 | 316,979 |
| SBLV MS18/KE/2019 | 1,262,464 | 6 | 5 | 106,632 | 2,265 | 21,241 |

**Table S8:** Sequencing statistics of native and capture-based target enrichment NGS for the M-segment of seven phleboviruses originating from sandfly samples.

| **Phlebovirus** | **No. of reads M-segment** | | | | | |
| --- | --- | --- | --- | --- | --- | --- |
|  | **Native** | | | **Enriched** | | |
|  | **Total reads** | **Viral reads** | **Viral reads per million generated reads** | **Total reads** | **Viral reads** | **Viral reads per million generated reads** |
| KBGV KS134/KE/2020 | 178 | 0 | 0 | 416,906 | 576 | 1,382 |
| BGRV MS21/KE/2019 | 1,068,724 | 50 | 47 | 636,216 | 192,066 | 301,888 |
| EMBV MS75/KE/2020 | 2,162,994 | 2 | 1 | 260,988 | 18,810 | 72,072 |
| SSKV MS134/KE/2020 | 21,299,276 | 143 | 7 | 3,574,752 | 109,203 | 30,548 |
| SSKV MS141/KE/2020 | 22,097,992 | 254 | 11 | 2,774,922 | 59,502 | 21,443 |
| SBLV SP109/KE/2019 | 408,130 | 42 | 103 | 242,628 | 13,172 | 54,289 |
| SBLV MS18/KE/2019 | 1,262,464 | 0 | 0 | 106,632 | 248 | 2,326 |

**Table S9:** Sequencing statistics of native and capture-based target enrichment NGS for the S-segment of seven phleboviruses originating from sandfly samples.

| **Phlebovirus** | **No. of reads S-segment** | | | | | |
| --- | --- | --- | --- | --- | --- | --- |
|  | **Native** | | | **Enriched** | | |
|  | **Total reads** | **Viral reads** | **Viral reads per million generated reads** | **Total reads** | **Viral reads** | **Viral reads per million generated reads** |
| KBGV KS134/KE/2020 | 178 | 0 | 0 | 416,906 | 16,371 | 39,268 |
| BGRV MS21/KE/2019 | 1,068,724 | 42 | 39 | 636,216 | 144,866 | 227,699 |
| EMBV MS75/KE/2020 | 2,162,994 | 12 | 6 | 260,988 | 30,982 | 118,710 |
| SSKV MS134/KE/2020 | 21,299,276 | 107 | 5 | 3,574,752 | 58,085 | 16,249 |
| SSKV MS141/KE/2020 | 22,097,992 | 155 | 7 | 2,774,922 | 26,440 | 9,528 |
| SBLV SP109/KE/2019 | 408,130 | 98 | 240 | 242,628 | 34,124 | 140,643 |
| SBLV MS18/KE/2019 | 1,262,464 | 0 | 0 | 106,632 | 448 | 4,201 |

**Table S10:** Average sequencing depth per segment of native and capture-based target enrichment NGS for seven phleboviruses originating from sandfly samples.

|  | **Average sequencing depth** | | | | | |
| --- | --- | --- | --- | --- | --- | --- |
| **Phlebovirus** | **L** | | **M** | | **S** | |
|  | **native** | **enriched** | **native** | **enriched** | **native** | **enriched** |
| KBGV KS134/KE/2020 | 0.0 | 587.0 | 0.0 | 19.7 | 0.0 | 1,334.4 |
| BGRV MS21/KE/2019 | 2.4 | 6,878.7 | 1.7 | 8,828.3 | 3.8 | 16,481.0 |
| EMBV MS75/KE/2020 | 0.1 | 840.3 | 0.0 | 739.1 | 0.9 | 2,788.9 |
| SSKV MS134/KE/2020 | 1.6 | 6,424.5 | 2.4 | 2,926.3 | 4.8 | 3,700.0 |
| SSKV MS141/KE/2020 | 2.6 | 3,652.8 | 4.5 | 1,600.3 | 7.4 | 1,821.5 |
| SBLV SP109/KE/2019 | 2.4 | 1,973.9 | 1.4 | 522.3 | 5.9 | 3,191.1 |
| SBLV MS18/KE/2019 | 0.1 | 56.9 | 0.0 | 9.5 | 0.0 | 41.7 |


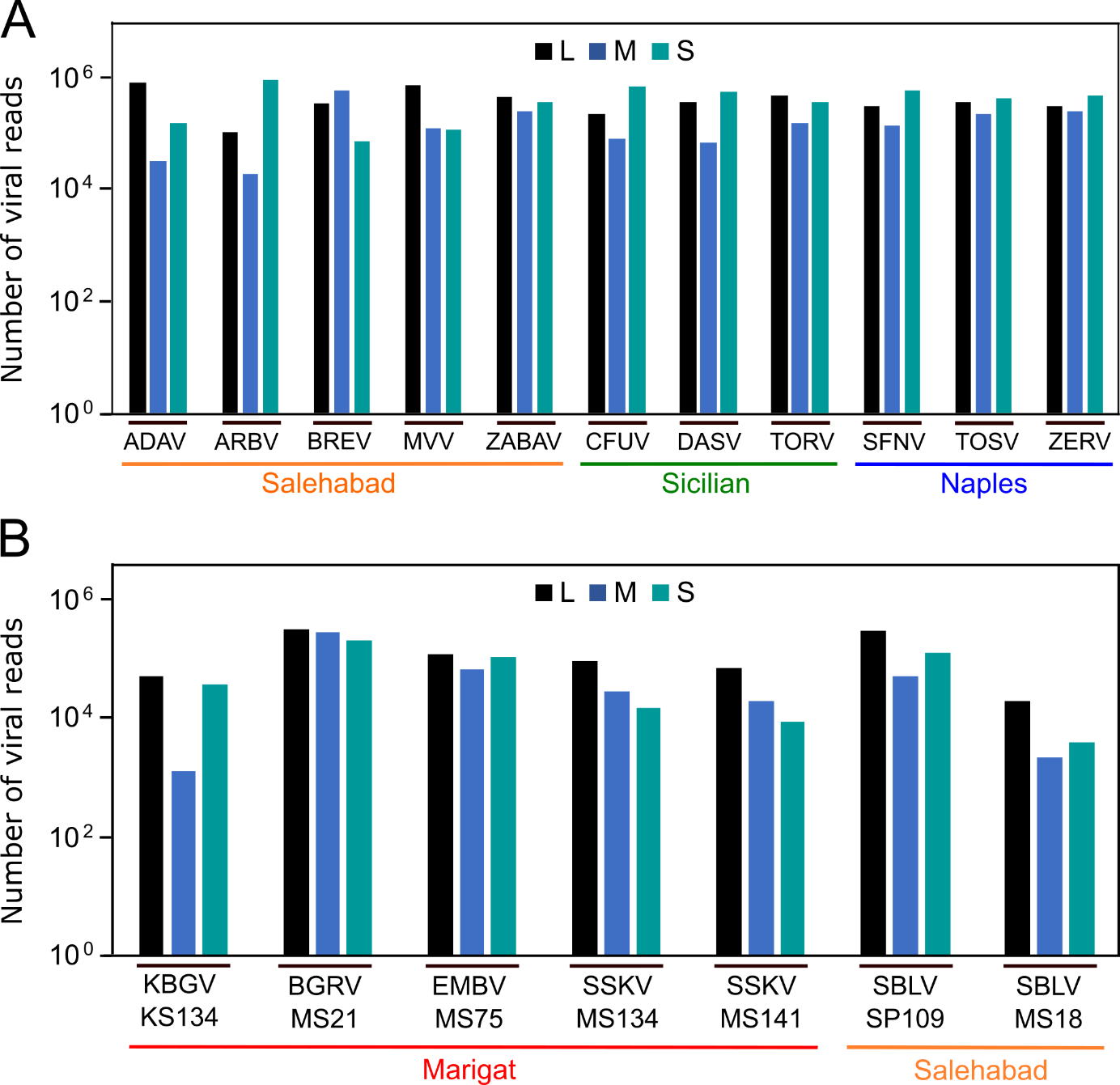


**Figure S1:** Performance of the bait set across genome segments. The number of viral reads per million generated reads after enrichment NGS are shown for A: phleboviruses from infectious cell culture supernatants, and B: phleboviruses from sandfly homogenates.
